# Investigation of an association between *in vitro* expression of *TMEM154* and *PARP14* genes and restriction of SRLV infection in primary skin cells of Carpathian goats

**DOI:** 10.2478/jvetres-2025-0072

**Published:** 2025-12-17

**Authors:** Magdalena Materniak-Kornas, Marlena Smagacz, Katarzyna Ropka-Molik, Aldona Kawęcka, Jacek Sikora, Jacek Michał Kuźmak

**Affiliations:** Department of Virology and Viral Animal Diseases, National Veterinary Research Institute, 24-100 Puławy, Poland; Department of Animal Molecular Biology, Department of Sheep and Goat Breeding, National Research Institute of Animal Production, 32-083 Balice, Poland; Department of Sheep and Goat Breeding, National Research Institute of Animal Production, 32-083 Balice, Poland

**Keywords:** small ruminant lentivirus, goat, restriction factor, skin culture, retrovirus, susceptibility to infection

## Abstract

**Introduction:**

Small ruminant lentivirus (SRLV) infections occur worldwide in goats and sheep and have negative impact on the production and welfare of animals. During recent years, many studies have focused on the host factors that determine the resistance of individual animals to SRLV infection; consideration of such factors would be an alternative to current control programmes based on culling seropositive animals. The aim of this study was to analyse the relationship between the expression of two previously selected goat genes, *TMEM154* encoding transmembrane protein 154 and *PARP14* encoding poly ADP-ribose polymerase 14, and the kinetics of SRLV replication in primary skin cells of goats. Potential role of these genes as host factors determining susceptibility to SRLV infection was investigated.

**Material and Methods:**

Primary fibroblast cultures obtained from the skin of goats with high SRLV proviral DNA load (HPL), low proviral load (LPL) or free of infection were inoculated with the A5 SRLV subtype circulating in the flock. The course of infection was observed based on cytopathic changes in cell cultures and the presence of SRLV A5 RNA, of which the level was monitored using a quantitative reverse-transcription PCR. The relative expression of the selected host genes following SRLV infection was analysed.

**Results:**

The kinetics of SRLV replication differed, and distinctly higher numbers of SRLV particles were detected in cells derived from the HPL animal. The expression profiles of *TMEM154* and *PARP14* after *in vitro* SRLV infection also differed in skin cells derived from HPL from the profiles in LPL-animal cells.

**Conclusion:**

The observed relationship between expression of *TMEM154* and *PARP14* and cell permissiveness after SRLV infection suggest their involvement in the infection process, but their utility as susceptibility factors still needs to be verified.

## Introduction

Small ruminant lentiviruses (SRLVs) are a group of viruses belonging to the *Retroviridae* family, comprising several genotypes (A, B, C, D and E) that infect sheep and goats (7). Genotypes A and B are responsible for most SRLV infections in the world, while the others are limited only to specific geographical regions. Historically, genotype A included strains similar to maedi-visna virus (MVV), and genotype B included strains similar to caprine arthritis-encephalitis virus (CAEV). These lentiviruses infect the monocytes and macrophages of sheep and goats, causing cross-infections and superinfections leading to chronic infection and wasting disease (20). In approximately 30% of infected goats, SRLV causes emaciation, progressive arthritis and mastitis, while in a similar percentage of sheep, it manifests as a progressive debilitating disease called maedi-visna. The main routes of SRLV transmission are the consumption of colostrum and milk from infected dams and prolonged direct contact between individuals (4, 25), while intrauterine transmission is considered a sporadic route of infection (2). Small ruminant lentivirus infections are widespread worldwide and have a significant impact on goat and sheep production and welfare (25). The most recent large-scale study performed by Kaba *et al*. (16) in 165 goat flocks in Poland showed 61% inter-herd and between 0.7% and 100% intra-herd level seroprevalence. As there is no effective vaccine against or treatment for SRLV infection, control programmes appear to be the only way to prevent the spread of the virus. The most effective practice is to cull animals revealed as infected by a positive serological test result. An alternative approach used in flocks with high seroprevalence is the selection of offspring from seronegative females and implementation of artificial feeding. Unfortunately, such practices have certain disadvantages, such as their ignorance of variation in serological response among different animals in flocks and of the virus’ high genetic variability, which complicates serological diagnosis (10, 27). In addition, they are costly and time-consuming, which is why it is necessary to introduce new, more effective control measures. In recent years, SRLV research has focused on host factors that determine the resistance of individual animals to SRLV infection. The identification of such markers may lead to the identification and selection of individuals that are naturally resistant to SRLV. This phenomenon is known for various pathogens like African swine fever virus or porcine reproductive and respiratory syndrome virus (1, 17), but the mechanisms specific to SRLV in sheep and goats are still under investigation.

The diagnosis of SRLV is mainly based on the detection of virus-specific antibodies, but new approaches include also PCR, which may not only detect provirus but also quantify its load. Unfortunately, no direct association between antibody titre and proviral load was confirmed in SRLV-infected animals because of the latency characteristic of lentiviral infections. However in several studies, high proviral loads of SRLV were linked with clinical manifestations of the disease (15, 26). Indeed, taking into account proviral load even in highly SRLV-infected flocks, individuals with low proviral load are considered those with limited ability to spread the virus (10). In consequence, low proviral load has been suggested as a biomarker of natural resistance to SRLV infection (10), but the exact mechanism behind that has not been revealed yet. Many recent studies aimed to identify the factors responsible for such resistance, but most of them were carried out in sheep. In order to fill this knowledge gap in goats, a Carpathian flock heavily infected with SRLV was investigated, revealing certain trends between specific single-nucleotide polymorphisms (SNPs) of several genes known as potential regulators of the immune response and the level of SRLV proviral DNA (18). Two genes, *PARP14* encoding poly ADP-ribose polymerase 14 and *TMEM154* encoding transmembrane protein 154, were suggested as potential markers for susceptibility or resistance to SRLV infection. Usually, population studies of the kind undertaken in goats are limited by factors of age, heterogeneity of infecting virus strains, different extents of exposure to the virus or individual host genetics, leading to confounding. Therefore, in order to understand the role of both genes in SRLV infection, an *in vitro* experiment was performed in controlled conditions. This study presents an analysis of virus replication kinetics as it associates with *TMEM154* and *PARP14* gene expression in primary skin cells from three goats after *in vitro* infection with A5 as the known SRLV subtype.

## Material and Methods

### Animals

The study was conducted in Carpathian goats from the National Research Institute of Animal Production Experimental Station in Odrzechowa with approval by the Local Ethical Committee on Animal Testing at the University of Life Sciences in Lublin, Poland. The goats were females at a similar age; all were healthy and kept in one herd in the same environment. The serological status of the animals for SRLV infection was confirmed by an ID Screen MVV/CAEV Indirect Screening Test ELISA (IDVet, Grabels, France) and the level of SRLV proviral DNA was determined as previously described (18).

### Isolation of SRLV subtype A5 in monocyte/macrophage culture

Fifty millilitres of blood was collected from several goats that were serologically positive for SRLV with high levels of proviral DNA. Monocytes were obtained from the blood by centrifugation on Histopaque. These cells were maintained in a differentiation medium, resulting in a macrophage culture after several days. After 7–10 d, when changes indicating viral infection (syncytia and cell lysis) appeared in the cultures, the culture medium was collected and centrifuged at 1,500 x *g* for 10 min and the obtained supernatant was frozen at -70°C for further analysis. The number of virus particles was determined by a quantitative reverse-transcription PCR (RT-qPCR) using primers specific for the A5 SRLV genotype (23).

### Preparation of cultures from goat skin samples

Skin fragments measuring 1 cm × 0.5 cm were collected from three selected goats: one uninfected (No. 8) and two classified as SRLV positive, one of which showed a high SRLV proviral load (HPL – No. 5) and the other a low proviral load (LPL – No. 19) in blood leukocytes. After collection, skin fragments were kept in phosphate-buffered saline (PBS) with antibiotics (Antibiotic-Antimycotic, Sigma-Aldrich, St. Louis, MO, USA) and then transferred to a 0.5% trypsin solution in the laboratory. Digestion was carried out at 37°C for about 40–50 min. The single cells and cell conglomerates obtained in this way were washed several times in warm PBS, then suspended in Dulbecco’s modified Eagle’s culture medium with 10% foetal calf serum and transferred to 25 cm^2^ culture flasks. The culture was maintained by changing the medium every 7 d until a monolayer of cells with fibroblast morphology was obtained.

### *In vitro* infection of skin cells with SRLV

In order to infect skin cells with SRLV, cultures were established in six-well plates loaded with 0.5 × 106 cells per well. When the cells formed a monolayer, they were infected by applying supernatant from infected goat macrophages containing a known amount of SRLV with a multiplicity of infection (MOI) of 1. The cells were washed after 15 h with PBS, and fresh culture medium was applied. Two replicates were established for each animal. Culture medium without virus was applied to the wells containing the control cultures. The cultures were maintained for 7 d, and 0.5 mL of culture fluid was collected every two days. After 7 d, the cells were scraped, washed with PBS and used for RNA isolation using RNeasy (Qiagen, Hilden, Germany). The culture medium from all cultures was centrifuged at 1,500 × *g* for 10 min and the obtained supernatant was also used for RNA isolation using the Viral RNA/DNA kit (EURx, Gdańsk, Poland).

### Quantitative PCR specific to the SRLV A5 subtype

Genomic DNA was isolated from goat peripheral blood leukocytes using the Blood Quick Pure test (Machery-Nagel, Düren, Germany). Proviral DNA of SRLV was quantified using qPCR with a Rotor-Gene Q thermocycler (Qiagen) and primers and a probe specific for the SRLV A5 subtype as described by Olech *et al*. (23), of which the presence in this herd had been previously confirmed (21). Amplification was performed in a total volume of 20 μL under the following conditions: initial incubation and polymerase activation at 95°C for 15 min, followed by 45 cycles at 94°C for 60 s and 60°C for 60 s. The reaction mixture contained 10 μL of 2× QuantiTect Probe PCR buffer (Qiagen, Hilden, Germany), 400 nM of each primer, 200 nM of specific probe and 5 μL of DNA template. All samples were tested in duplicate, and the results were expressed as the average number of copies per 500 ng of genomic DNA from each goat, based on a standard curve determined from reactions containing 10 serial dilutions (from 108 to 100 copies) of plasmid DNA carrying a 625-base-pair fragment of the SRLV A5 *gag* gene. The same method was used to determine the level of viral RNA in the cell-culture supernatants from macrophages and skin cells, but then 2 μL of complementary DNA (cDNA) was used as the template.

### Reverse transcription

A reaction was performed to obtain cDNA for further analysis (RT-qPCR and gene expression analysis) using the NGdart kit (EURx, Gdańsk, Poland) for 60 min at 50°C. The RNA used in this reaction was isolated as described above and was treated with DNase I (Life Technologies, Carlsbad, CA, USA) for 15 min at room temperature prior to the RT reaction. The reaction was stopped by adding ethylenediaminetetraacetic acid and incubating the mixture for 10 min at 65°C.

### Gene expression analysis

Relative gene expression was investigated using an RT-qPCR. Total RNA was extracted from skin cells using the RNeasy RNA Extraction Kit (Qiagen). The concentration and integrity of the RNA were measured using a TapeStation system (Agilent Technologies, Waldbronn, Germany). Complementary DNA was synthesised from 0.5 μg of RNA as described above. The assay was performed using QuantiTect SYBR Green Mastermix (Qiagen, Hilden, Germany) in a C1000 Touch thermocycler and CFX383 PCR Detection System (Bio-Rad, Hercules, CA, USA) using primers specific for the *TMEM154* and *PARP14* genes and for *GAPDH* as a house-keeping gene (Table 1). Amplification of each gene was performed in a total reaction mixture volume of 25 μL containing 12.5 μL of 2× QuantiTect SYBR Green PCR buffer (Qiagen, Hilden, Germany), 400 nM of each primer, 200 nM of a specific probe and 1 μL of cDNA template. The reaction temperature profile was initial incubation and polymerase activation at 95°C for 15 min followed by 45 cycles of denaturation at 94°C for 60 s, primer annealing at 55°C for 30 s and elongation at 72°C for 30 s. Finally, the melting temperatures of the PCR products were analysed. All samples were tested in two replicates. The yield of each reaction was determined based on a standard curve established from 10-fold dilutions of the cDNA mixture from all tested samples (from 100 to 1). The transcription level of each gene was determined as a relative value to the housekeeping gene according to the ΔΔCt method and presented as FC (fold change). In such analyses, it is assumed that an FC value above 1.5 indicates upregulation of gene expression, while an FC value below 0.6 indicates its downregulation.

**Table 1.** Primers used in the analysis of gene expression

Gene	Sequence of the primer (5'-3')	Orientation	Fragment length	Reference
*GAPDH*	TTCTGGCAAAGTGGACATCGT	F	112 bp	(19)
	CTTGACTGTGCCGTTGAACTTG	R		
*TMEM154*	ATTTCTCTGTCACCTGGCCA	F	155 bp	(18)
	AGACAGCAAACAAAGCAAGTATT	R		
*PARP14*	CGGGTACTCACTGGATGCTA	F	203 bp	(18)
	TCTGCAAAGGTTACCAAAATGTT	R		

1 *GAPDH* – Glyceraldehyde 3-phosphate dehydrogenase; *TMEM154* – Transmembrane protein 154; *PARP14* – Poly ADP-ribose polymerase 14; F – forward; R – reverse

## Results

The study was conducted in a flock of Carpathian goats with the history of SRLV infection, in which previous studies had found only one subtype, A5 (21). Selection of goats with HPL and LPL was made previously (18). For this study, in order to isolate the SRLV A5 subtype circulating in this flock, blood samples were collected from the two goats with the highest level of proviral DNA. Because macrophages are the main target cells of SRLV, monocytes were derived from the blood and differentiated into macrophages by culturing them in a differentiation medium. The supernatant was collected from the macrophage cultures and the virus level was determined by RT-qPCR, yielding a high titre of approximately 5×10^8^ SRLV particles per mL. In parallel, primary skin cell cultures, predominantly fibroblasts, were obtained from skin fragments of three goats, one from an HPL animal (goat No. 5), one an LPL animal (goat No. 19), and the last an uninfected control animal (goat No. 8). All three primary skin cell sets were infected with the previously obtained inoculum containing free SRLV subtype A5 virus at a ratio of 1 : 1 (MOI 1). Part of the cells from each animal were left as the controls.

**Fig. 1.**
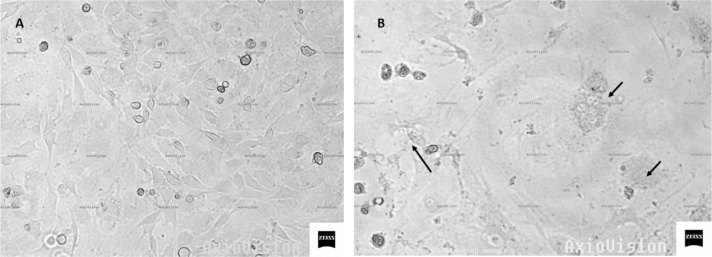
Photomicrographs of skin cells: A – uninfected cells; B – cultured cells at 7 days after SRLV infection (arrows indicate syncytia)

The course of infection was observed based on changes in the cell culture, noting the appearance of a cytopathic effect in all infected cultures, while no typical syncytia were observed in uninfected cultures. It should be noted, however, that the lytic process was particularly strong in cells derived from goat No. 5, from the HPL group. Example images of an infected and uninfected culture are shown in Fig. 1.

**Fig. 2.**
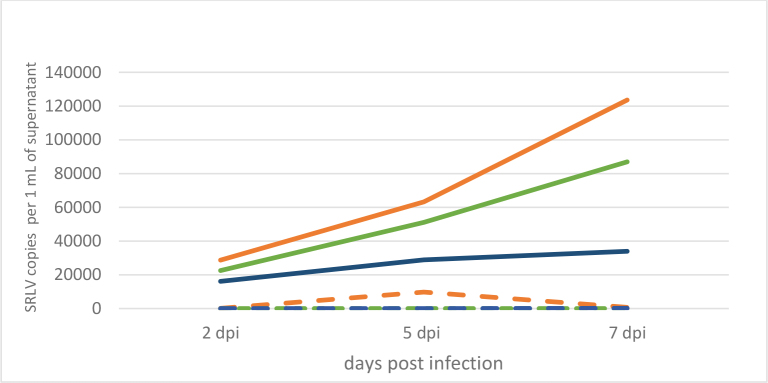
Kinetics of SRLV replication in experimentally infected skin cells from a high-proviral-load goat (solid orange line), a low-proviral-load goat (solid blue line) and an uninfected goat (solid green line) and these kinetics in cells left without experimental infection from the same goats (dashed lines)

Extraction of RNA was achieved from the culture medium of infected and uninfected cells collected regularly, and this genetic material was used as a template in the RT-qPCR reaction, in order to monitor the kinetics of SRLV replication in cells derived from individual animals (Fig. 2). Interestingly, the largest increase of SRLV was shown in cells from the HPL goat – from around 30,000 particles per mL at 2 days post infection (d.p.i) to over 120,000 at 7 d.p.i., while in the cells of the LPL goat only a twofold increase in the number of viral particles was observed between 2 d.p.i. (16,000) and 7 d.p.i. (33,000). A second interesting observation was the effective replication of SRLV in the cells of the uninfected goat, similar to that observed in the cells of the HPL goat, but less proliferatively, from 22,000 particles at 2 d.p.i. to 86,000 at 7 d.p.i.

**Fig. 3.**
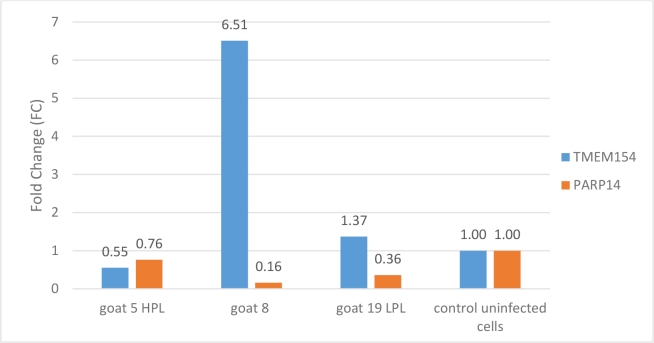
Relative gene expression in skin cells of goats Nos 5, 8 and 19 experimentally infected with SRLV and in these cells from all three animals left without experimental infection as controls. HPL – high proviral load; LPL – low proviral load; *TMEM154* – transmembrane protein 154; *PARP14* – poly ADP-ribose polymerase

The third element examined in this study was the relative expression of the *TMEM154* and *PARP14* host genes in the skin cells of the three goats after SRLV infection as well as in uninfected cells. Notably, the expression of the *TMEM154* gene was significantly increased (FC = 6.5) after SRLV infection in skin cells from goat No. 8, *i.e*. the uninfected animal, while the expression of the *PARP14* gene was clearly decreased (Fig. 3). A similar trend of both genes’ expression was observed in skin cells from goat No. 19 with an LPL. Interestingly, in skin cells from goat No. 5 with an HPL, the expression of the *TMEM154* gene was clearly weaker (FC = 0.55), while the expression of the *PARP14* gene was only slightly lower than it was in uninfected cells (FC = 0.76).

## Discussion

The study aimed to analyse the expression of *TMEM154* and *PARP14* genes in primary skin cells of Carpathian goats following infection with the A5 subtype of SRLV. Monocytes and monocyte-derived macrophages are the main targets for SRLV, and both types of cells express viral proteins, triggering specific and unspecific immunological responses (9). However, only some infected individuals develop disease symptoms, while the remaining ones are latently infected. It has been suggested that higher viral load predisposes individuals to the development of disease (13, 26), while low proviral load is a consequence of some kind of restriction. Such LPL animals develop the serological response, but viral replication in them is relatively low, and the risk of their spreading the virus within the flock is diminished. Therefore, such LPL animals are called non-progressors (28). Unfortunately, this phenomenon is disregarded in control programmes, which still are based only on serological confirmation of the infection and removal of the seropositive animals (6). Therefore, in recent years scientists have been making efforts to reveal the specific mechanisms underlying observed restriction in LPL animals. Understanding these mechanisms would be extremely valuable in the disease control and eradication in highly infected flocks, in which current control programmes are problematic, because they recommend culling all seropositive individuals. Different possible mechanisms of resistance to SRLV infections have been proposed, including involvement of already known restriction factors such as APOBEC3 (apolipoprotein B editing complex 3), Trim5α (tripartite motif protein 5 alpha), or Tetherin, which hinders the replication of lentiviruses when overexpressed (10). Other theoretical determinants are specific SNPs in the genes encoding the restriction factors referred to, as well as in *TMEM154, TLRs* (Toll-like receptors), *MYD88* (myeloid differentiation primary response 88 innate immune signal adapter) and *CCR5* (chemokine (C-C motif) receptor 5), as factors responsible for influencing the functions of their encoded proteins in terms of virus–host interactions (3, 13, 29). Most studies were carried out in sheep, while only a few reports focused on the identification of SRLV resistance markers in goats (8, 22, 23).

**Fig. 4.**
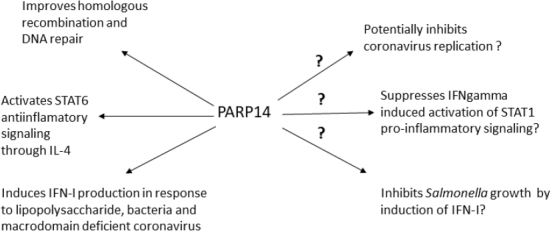
Known and probable functions of the PARP14 protein. STAT – signal transducer and activator of transcription; IFN – interferon; IL-4 – interleukin 4

In our previous study we already identified certain trends in the correlation of specific SNPs of several goat genes, reported previously as potential regulators of the immune response, with the level of SRLV proviral DNA (18). Therefore, in the present study we aimed to extend these observations and focus on the examination of expression of *TMEM154* and *PARP14* following SRLV infection in cells from HPL, LPL and uninfected goats. In addition we investigated the kinetics of SRLV replication. All cells were productively infected with the SRLV strain circulating in this flock, classified as A5 subtype, but the highest replication of the virus and respective cytopathic effect was observed in cells derived from the HPL goat, while the virus multiplied least in cultures from LPL-goat cells, which suggests the functioning of certain restriction mechanisms. However, SRLV also efficiently replicated in the skin cells of the uninfected goat No. 8, which was quite unexpected. In our previous report (18), the TT genotype in the *PARP14* locus was observed only in seronegative individuals, while the *TMEM154* CC genotype was observed only in goats infected with SRLV. It means that goat No. 8, which was not infected with SRLV, carried a different genotype of both genes to the type carried by the HPL and LPL goats. Our observations suggest that the particular *PARP14* and *TMEM154* genotype did not affect the permissiveness of skin cells in the *in vitro* setting, as virus replication was quite efficient, although lower than in the cells of the HPL goat. In this context, the differences in the expression of both genes in the skin cells of individual goats seem to be interesting. We showed that the expression of *TMEM154* was upregulated in the skin cells of the LPL animal and those of the uninfected goat, but at different levels, while in the HPL goat’s cells it was clearly downregulated. The opposite expression was observed for *PARP14*. It was downregulated in the skin cells of the LPL goat and the uninfected individual, while in the HPL goat its expression was only slightly weaker and was not defined as downregulation. The *TMEM154* gene has been described several times in the context of numerous polymorphisms found in sheep, some of which appear to be significantly related to the susceptibility of these animals to SRLV infection. Research by Heaton *et al*. (14) has shown that sheep with haplotype 3, encoding glutamic acid at position 35 and asparagine at position 70 of the TMEM154 protein, and haplotype 2, encoding isoleucine at position 70 of this protein, are highly susceptible to SRLV infection. In contrast, individuals with haplotype 1, encoding lysine at position 35 of the protein, are considered to be much less susceptible. In goats, we did not find the same polymorphisms, but a different one (TMEM154, T>C, 17: 65805153), with the CC genotype observed only in SRLV-infected animals (18). Although *TMEM154* is often described in the context of resistance to SRLV infection, its function has not been established yet. Knowledge of the functions of the *PARP14* gene’s protein is much more advanced (Fig. 4) (24). Surprisingly, our observations regarding *PARP14* were contrary to the results of experiments performed previously on human cells, which showed that the PARP14 protein induces type I interferon production in response to bacterial and viral infections and its gene is often identified among genes with increased expression in viral infections (5, 11, 12). It is possible that the inconsistency of the results was due to the stage of the experiment (7 days after infection) or the type of cells used. Of course the species from which the cells originated may also influence the result. Despite the productive infection of all skin cells derived from the three goats, the replication potential was clearly lower in the LPL goat and the originally uninfected one. Furthermore, these two goats also shared a similar expression profile of *TMEM154* and *PARP14*, which differed to that of the HPL individual. Although our study was limited to a few selected animals, our findings confirm involvement of the *TMEM154* and *PARP14* in limiting SRLV replication.

## Conclusion

Identifying the relevant genes and their polymorphisms associated with goat susceptibility to infection is an important step toward reducing SRLV transmission in sheep and goats. The presented study demonstrated differences in the permissiveness of goat skin cells with different polymorphisms to SRLV infection and differences in their expression. The results obtained suggest that selected genes may be involved in the control of SRLV infection, but their utility as indicators of SRLV infection susceptibility requires further analysis in a larger group of goats.
